# Hydration behavior of L-proline in the presence of mono, bis, tris-(2-hydroxyethyl) ammonium acetate protic ionic liquids:  Thermophysical properties

**DOI:** 10.1038/s41598-024-77341-6

**Published:** 2024-11-08

**Authors:** Mohammad Amin Morsali, Hemayat Shekaari, Behrang Golmohammadi

**Affiliations:** Department of Physical Chemistry, Faculty of Chemistry, University of Tabriz, Tabriz, Iran

**Keywords:** Hydration behavior, L-proline, Protic ionic liquids, Volumetric properties, Viscosity *B*-coefficient, COSMO, Thermodynamics, Density functional theory, Statistical mechanics

## Abstract

The hydration behavior of amino acids, essential for biological macromolecules, is influenced by ammonium biomaterials. The protic ionic liquids (PILs) are gaining attention in the food and pharmaceutical industries due to their nontoxicity and adjustable properties. Thus, study of the amino acids, such as L-proline, in the presence of PILs is crucial for understanding their hydration behavior. In this work, the effect of PILs, including mono, bis, tris (2-hydroxyethyl)ammonium acetate protic ionic liquids that might be naturally produced in human body, on L-proline hydration behavior was studied using COSMO calculations and thermophysical measurements. Measurements were the density, speed of sound, viscosity, and refractive index data of the solutions (L-proline + PILs + water) at various PIL concentrations at temperatures (298.15 to 318.15) K and under atmospheric pressure. The study indicates L-proline has weaker interactions with water compared to PILs ([2-HEA][Ac], [bis-2-HEA][Ac], and [tris-2-HEA][Ac]) due to its compact structure and lower negative dielectric energy. PILs interact more strongly with water through hydrogen bonding. Increasing temperature affects L-proline’s hydration layer, releasing more water molecules compared to PIL solutions. This effect is more pronounced with [tris-2-HEA][Ac], likely due to its larger size and complex structure. While L-proline promotes an ordered water structure, PILs can disrupt this by rearranging water molecules and forming their own hydrogen bonds.

**Supplementary Information:**

The online version contains supplementary material available at 10.1038/s41598-024-77341-6.

## Introduction

Water, intricately bound to biopolymers like proteins and DNA through hydrogen bonding (H-bonding), acts as a silent maestro in their function and stability within living organisms^1^. H-bonding synchronizes the structure and behavior of water in various contexts, from liquid water and ice to solvation shells, protein folding, and DNA replication^2^. The amphoteric nature of amino acids, the building blocks of proteins, has garnered significant interest due to their ability to form extensive H-bonds with surrounding water molecules^3^. These relatively simple biomolecules serve as model systems to decipher the intricate physicochemical interactions in more complex biological structures like enzymes and protein assemblies^4,5^. To unravel the nature of H-bonding and hydration in aqueous environments, researchers employ various tools, especially spectroscopic techniques^4,5^. These methods not only reveal hydration properties but also offer structural insights by analyzing H-bonds within the system. Vibrational spectroscopy, encompassing infrared, near-infrared, and Raman techniques, is a well-established approach for detecting and quantifying H-bonding in simple and complex molecules^6^.

A comprehensive investigation was conducted into the equilibrium solubility and thermodynamic behavior of L-proline in aqueous and aqua-electrolytic solutions, focusing on the effects of varying concentration and temperature, and utilizing analytical gravimetric techniques to explore chemical interactions and the salting in/out effect^7^. The dielectric permittivity of L-proline in water and ethanol solutions was measured using an open-ended coaxial probe technique, revealing higher dielectric relaxation times in ethanol due to stronger self-association and hydrogen bonding, with results supported by DFT/B3LYP and MP2 calculations^8^. The influence of cis/trans proline isomerization on the phase behavior of elastin-like polypeptides (ELPs) was explored, showing that proline isomerization tunes the conformational behavior without altering the transition temperature^9^. Multi-wavelength UV resonance Raman spectroscopy was employed to study hydrogen bonding around the proline-based tripeptide glycyl-L-prolyl-glycinamide·HCl, revealing a strong ion-peptide interaction effect on conformational stability, particularly in the presence of fluoride and chloride anions^10^.

Protic ionic liquids (PILs) have captured global interest as versatile designer solvents for diverse applications^11^. Their appeal stems from a unique blend of advantageous properties. These properties arise from intricate networks of cooperative non-covalent interactions, particularly hydrogen bonding (H-bonding)^3^. Researchers have extensively studied the impact of H-bonding strength, directionality, and location between PIL cations and anions on their pure state properties^12,13^. However, a gap exists in our understanding of PIL-water interactions, particularly regarding non-covalent interactions. This knowledge gap is crucial as PIL-water mixtures are increasingly used for dissolving, recrystallizing, and stabilizing biomolecules. Carboxylate anion hydration holds particular significance in biochemistry and solution chemistry. This functional group is found in biomolecules such as proteins and amino acids^14,15^. Notably, enzyme active sites often involve carboxylate groups for ligand binding. Investigating simpler model systems like ethanolamine carboxylate, where both cations and anions are present in biological systems, offers a more accurate reflection of their behavior.

To design and improve the biotechnological processes, the information about the molecular mechanism interactions existing between pharmaceutically active ionic liquid form and biomolecules is required^16^. Protein is intrinsically complicated molecule which made up of amino acids so the studying of amino acids is much easier than proteins. In this regard, thermophysical properties of amino acids in the presence of protic ionic liquids with water give us effective information about the solute-solvent and solute-solute interactions^17^. Over the last few years, numerous scientists have conducted experiments to measure the thermodynamic properties of mixtures containing amino acids and first-generation ionic liquids in aqueous solutions at different temperatures^18,19^. Denaturation of amino acids made us to study on thermodynamics properties of amino acids with water in the presence of protic ionic liquids and protective effect of these special solvents on denaturation of amino acids^20,21^.

As clear from the related works, there are significant amount of spectroscopic and computational investigations that declare the H-bonding, and the possible interaction between the water and amino acids in the presence of PILs. On the other hand, these data should be approved by thermophysical properties behavior of these systems. In this respect, there are just few methods that could relate spectroscopic results that have been proved by quantum mechanics with thermophysical results through statistical mechanic. One of the most important and modern statistical mechanic models is COSMO model that could be used to understand the thermophysical behavior through quantum mechanics results.

In this work, the interactions between the L-proline and PILs (2-hydroxyethylammonium acetate, Bis-(2-hydroxyethylammonium acetate), Tris-(2-hydroxyethylammonium acetate) in aqueous media have been investigated. The density, speed of sound, viscosity and refractive index of aqueous L-proline in the presence of different concentration of the PILs were measured at (288.15 k to 318.15) K and atmospheric pressure. The measured data were used to compute partial molar volume ($V_{\varphi }^{0}$) partial molar isentropic compressibility ($\kappa _{\varphi }^{0}$) viscosity *Ɓ*-coefficient and molar refraction *R*_*M*_. Also, the COSMO calculation has been carried out to understand the bonding and interactions between the L- proline and the ionic liquids in the term of σ-profiles and COSMO results.

## Materials and methods

### Materials

All reagents including L-proline, ethanolamine, di-ethanolamine, triethanolamine, and acetic acid have been purchased from Merck, and used without further purification. Also, deionized ultrapure water with a specific conductance below 1µS cm^− 1^ was used to prepare the corresponding aqueous solutions of amino acid in the presence of ionic liquids. The information of the utilized materials is given in Table 1.

**Table 1:** Sample description of pure materials.

Chemical Name	Abbreviation	CAS. NO	Source	Purity w/w%	Purification Method	Analysis methods
*L*- Proline	-	147-85-3	Merck	> 0.99	–	–
2-Hyroxtethylammonium acetate	[2-HEA][Ac]	54300-24-2	Synthesized	> 0.98	Rotary evaporation	^1^H NMR, FT-IR, Karl-Fischer
Bis-(2-hydroxyethylammonium) acetate	[bis-2-HEA][Ac]	23251-72-1	Synthesized	> 0.98	Rotary evaporation	^1^H NMR, FT-IR, Karl-Fischer
Tris-(2-hydroxyethylammonium) acetate	[tris-2-HEA][Ac]	14806-72-5	Synthesized	> 0.99	Rotary evaporation	^1^H NMR, FT-IR, Karl-Fischer

### PILs synthesis and purifications

In this research, the ionic liquids used, including 2-hydroxyethylammonium acetate, bis-(2-hydroxyethylammonium) acetate, and tris-(2-hydroxyethylammonium) acetate were synthesized and purified. Briefly, acetic acid was added dropwise to ethanolamines (mono-ethanolamine, di-ethanolamine, and tri-ethanolamine) that placed in an ice bath and stirred vigorously with a magnetic stirrer at room temperature for 24 h. In order to achieve higher purity, the obtained ionic liquid is dried for 3 h with a vacuum pump. In this regard, a vacuum pump model D25 made in America was used for desiccation. The PILs have been characterized by ^1^H-NMR and FT-IR spectra besides the Karl-Fisher analysis to determine the water content. These results have been given in supporting information.

### Apparatus and procedure

The solution for investigating the volumetric, acoustic, and transport properties of the studied systems was prepared using the Shimadzu Aw-220 analytical balance, which has an uncertainty of ± 2 × 10^− 4^g. To measure the density and speed of sound, a digital vibrating U-shaped densitometer (Anton Paar DSA5000) with a resolution of ± 1 × 10^− 6^ g cm^− 3^ and 0.01 m s^− 1^ respectively was used. The uncertainty in measuring these properties was 4 × 10^− 5^ g cm^− 3^ and 0.7 m s^− 1^. The instrument was calibrated using air and distilled water, and the frequency for measuring the speed of sound was 3 MHz. A digital micro viscometer (Rolling-Ball Viscometer: Lovis 2000 M) was used to measure the viscosity of the solutions, calibrated at room temperature (298.15 K) using doubly distilled water. The Digital Refractometer (Abbe refractometer- ATAGO) instrument, which has an accuracy of approximately ± 0.0002 units, was used to measure the refractive index. The refractometer was calibrated twice using doubly distilled water to measure the refractive index. A DFT geometry optimization of the PILs have been carried out by Dmol^3^, generalized gradient approximation- Vosko- Wilk- Nusair- BP (GGA VWN-BP) which BP functional has been implemented for replacing local correlation in VWN functional to obtain COSMO results in two step including geometry optimization and energy optimization.

## Results and discussion

### Molecular fingerprint: σ-profile

The sigma-profile is a crucial concept in COSMO-based thermodynamics, representing the charge distribution on a molecule’s surface. It acts as a unique fingerprint, indicating the likelihood of finding specific charge density values in segmented segments. COSMO models, such as COSMO-RS and COSMO-SAC, utilize sigma-profiles to predict thermodynamic properties and molecule-environment interactions. These profiles are obtained through computational methods, primarily employing density functional theory (DFT) calculations, which can be computationally expensive. To overcome this, alternative methods approximating sigma-profiles are available in software tools and databases, enabling faster analysis, especially in high-throughput screening applications.

**Fig. 1:**
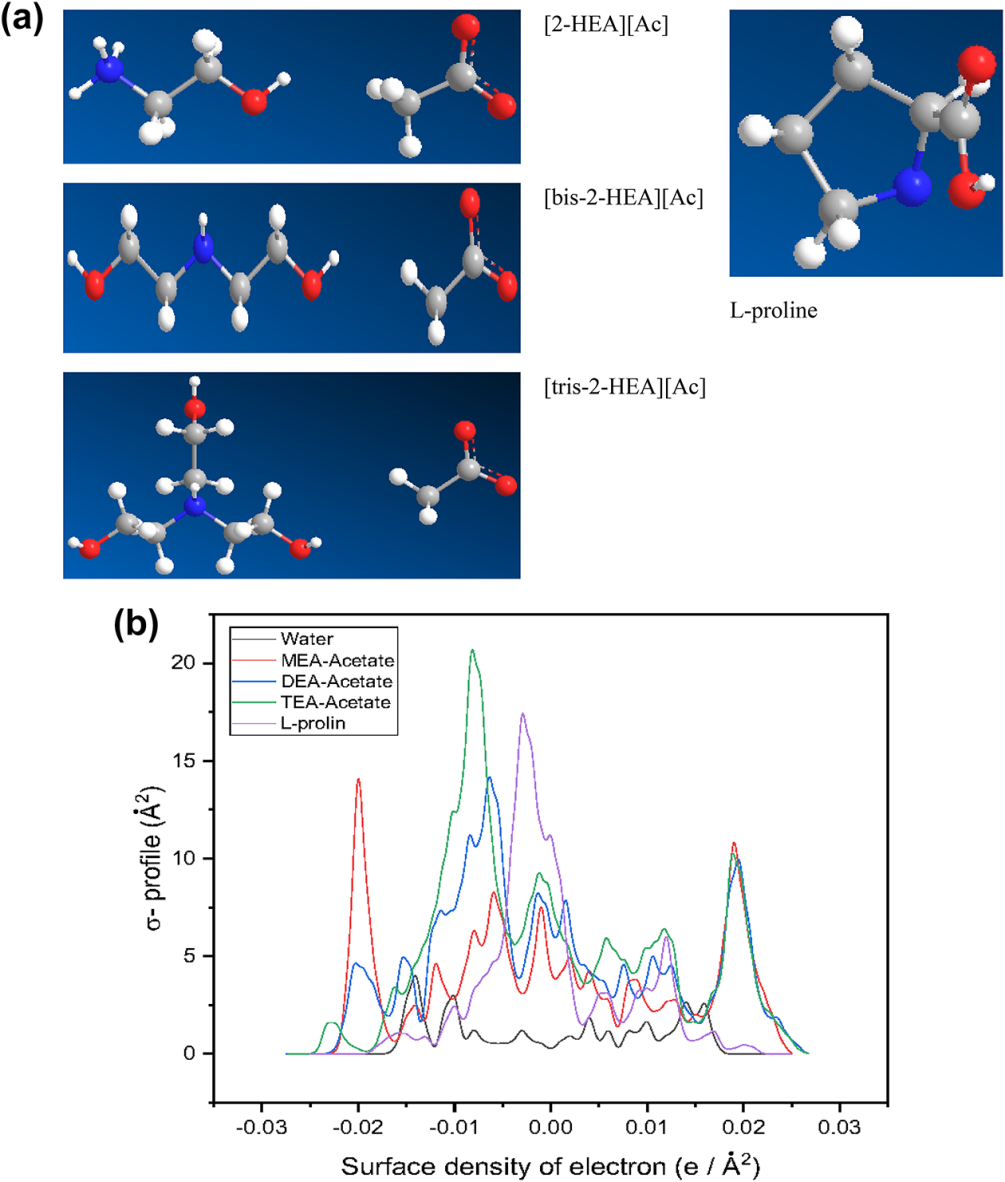
(**a**) Molecular structure of the studied compounds, (**b**) Sigma profiles of L-proline, water, MEA Acetate, DEA Acetate, and TEA Acetate.

The utilization of the GGA VWN-BP function in Dmol^3^, as suggested by the developer, has shown promising results for real solvents. In this study, COSMO results were obtained through DFT calculations using the Dmol^3^ module of Materials Studio (Biovia, Materials Studio 2023). The molecule geometry was optimized using GGA (VWN-BP). Figure 1 presents the optimized structures of the molecules and the corresponding COSMO results, specifically the σ-profiles, for the solvents and IL under investigation.

Additionally, Table 2 provides the cavity volume and cavity surface area results besides the HOMO and LUMO energy of the compounds from Dmol^3^ energy optimization calculations. The GGA VWN-BP function is a crucial exchange-correlation functional used in DFT calculations. Specifically, it is employed in the Dmol^3^^3^ module of Materials Studio for electronic structure calculations and simulations.

**Table 2:** The cavity volume and surface beside hydration energy, and highest occupied molecular orbital numbers and energy, lowest unoccupied molecular orbital of the studied compounds.

Compound	Cavity volume	Cavity surfacer	Dielectric (hydration) energy	*n*_HOMO_	HOMO	*n*_LUMO_	LUMO
	Å^3^	Å^2^	kcal/mol		eV		eV
L-proline	132.672	144.705	−11.98	31	−5.407 eV	32	−1.079 eV
[2-HEA][Ac]	155.249	188.538	−114.85	33	−4.457 eV	34	0.498 eV
[bis-2-HEA][Ac]	207.557	241.680	−111.79	45	−4.439 eV	46	0. 226 eV
[tris-2-HEA][Ac]	251.179	271.476	−103.36	57	−4.444 eV	58	−0.047 eV

This function combines the exchange functional of Vosko, Wilk, and Nusair with the correlation functional developed by Becke and Perdew. The exchange part facilitates electron exchange between orbitals, while the correlation part accounts for electron repulsion or attraction resulting from their interactions. By integrating these components, the VWN-BP function approximates the exchange-correlation effects in the system. Belonging to the class of generalized gradient approximation (GGA) functionals, it considers not only the electron density but also its gradient, enabling a more accurate representation of the system’s electronic structure compared to simpler functionals like the local density approximation (LDA). In Dmol^3^, the GGA VWN-BP function serves to optimize molecular geometry, compute electronic properties, and perform various DFT calculations. Its utilization aims to deliver reliable and precise outcomes across diverse applications, including the examination of solvation behavior in real solvents. In all of the cases in this study water has been chosen for solvent.

L-proline has the smallest cavity volume and surface area compared to the other molecules, suggesting a more compact solute. It also has the least negative dielectric (hydration) energy, indicating a weaker interaction with the solvent compared to the other molecules. [2-HEA][Ac], [bis-2-HEA][Ac], and [tris-2-HEA][Ac] have progressively larger cavity volumes and surface areas, implying they occupy more space and have more intricate interactions with the solvent compared to L-proline. They also exhibit more negative dielectric (hydration) energies, signifying stronger favorable interactions with the solvent. The HOMO energies for all molecules are negative, and the LUMO energies vary across the compounds.

### Volumetric properties

The density of the ternary aqueous solution of the L-proline in the presence of [2-HEA][Ac], [bis-2-HEA][Ac], and [tris-2-HEA][Ac] under atmospheric pressure (*P =* 0.086 MPa) and temperature range of (288.15− 318.15) K are presented in Table 3.

**Table 3:** The density of ternary solutions containing L-proline in aqueous PILs and apparent molar volume of L-proline in various concentration of PILs and temperature range of (288.15-318.15) K under atmospheric pressure of (0.086 MPa).

*m*	*d* / g cm^− 3^				10^6^ *V*_*φ*_ / m^3^ mol^− 1^			
	288.15 K	298.15 K	308.15 K	318.15 K	288.15 K	298.15 K	308.15 K	318.15 K
[2-HEA][Ac]: 0.05 mol·kg^− 1^								
0.0000	1.000887	0.998202	0.995635	0.993323				
0.0497	1.002513	0.999793	0.997196	0.994870	82.21	83.05	83.79	84.20
0.0993	1.004126	1.001362	0.998729	0.996397	82.19	83.12	83.93	84.25
0.1496	1.005746	1.002950	1.000285	0.997927	82.21	83.09	83.89	84.33
0.2001	1.007378	1.004525	1.001798	0.999455	82.12	83.10	84.05	84.33
0.2499	1.008958	1.006065	1.003308	1.000934	82.13	83.11	84.02	84.40
0.2992	1.010504	1.007574	1.004766	1.002397	82.16	83.12	84.08	84.40
[2-HEA][Ac]: 0.10 mol·kg^− 1^								
0.0000	1.002180	0.999470	0.996889	0.994575				
0.0494	1.003780	1.001028	0.998419	0.996058	82.51	83.50	84.21	85.29
0.0997	1.005417	1.002623	0.999977	0.997561	82.29	83.27	84.06	85.22
0.1498	1.007050	1.004216	1.001522	0.999056	82.11	83.08	83.98	85.14
0.1993	1.008701	1.005819	1.003061	1.000553	81.78	82.78	83.82	84.93
0.2496	1.010330	1.007424	1.004612	1.002051	81.70	82.63	83.70	84.84
0.2994	1.012022	1.009066	1.006181	1.003537	81.35	82.31	83.48	84.73
[2-HEA][Ac]: 0.15 mol·kg^− 1^								
0.0000	1.003469	1.000732	0.998139	0.995820				
0.0501	1.005048	1.002267	0.999645	0.997316	83.32	84.34	85.06	85.39
0.1005	1.006655	1.003830	1.001170	0.998826	82.98	84.00	84.81	85.19
0.1501	1.008242	1.005372	1.002677	1.000322	82.76	83.79	84.61	84.98
0.1993	1.009851	1.006926	1.004202	1.001821	82.41	83.50	84.30	84.75
0.2500	1.011542	1.008530	1.005770	1.003367	82.00	83.25	84.07	84.53
0.3002	1.013202	1.010112	1.007309	1.004894	81.74	83.07	83.92	84.37
[bis-2-HEA][Ac]: 0.05 mol·kg^− 1^								
0.0000	1.001325	0.998629	0.996050	0.993730				
0.0500	1.002960	1.000207	0.997590	0.995241	82.24	83.52	84.42	85.14
0.1004	1.004595	1.001789	0.999125	0.996752	82.23	83.47	84.46	85.12
0.1494	1.006172	1.003315	1.000610	0.998232	82.23	83.45	84.44	84.96
0.1998	1.007782	1.004865	1.002135	0.999743	82.21	83.47	84.37	84.86
0.2489	1.009348	1.006367	1.003612	1.001209	82.18	83.47	84.33	84.79
0.3000	1.010972	1.007916	1.005141	1.002728	82.12	83.48	84.28	84.72
[bis-2-HEA][Ac]: 0.10 mol·kg^− 1^								
0.0000	1.003074	1.000320	0.997743	0.995078				
0.0498	1.004691	1.001905	0.999295	0.996598	82.41	83.19	83.99	84.78
0.1001	1.006315	1.003504	1.000847	0.998139	82.33	83.04	83.98	84.56
0.1496	1.007885	1.005065	1.002379	0.999651	82.41	83.00	83.87	84.43
0.1998	1.009483	1.006635	1.003921	1.001183	82.36	82.98	83.81	84.32
0.2494	1.011054	1.008175	1.005402	1.002681	82.33	82.97	83.90	84.28
0.2994	1.012581	1.009701	1.006926	1.004207	82.44	83.01	83.81	84.14
[bis-2-HEA][Ac]: 0.15 mol·kg^− 1^								
0.0000	1.004703	1.001924	0.999297	0.996945				
0.0498	1.006317	1.003494	1.000838	0.998448	82.38	83.40	84.12	85.01
0.1004	1.007947	1.005084	1.002397	0.999978	82.31	83.28	84.02	84.82
0.1501	1.009539	1.006632	1.003924	1.001481	82.28	83.27	83.95	84.69
0.1997	1.011099	1.008161	1.005435	1.002971	82.34	83.28	83.92	84.61
0.2495	1.012678	1.009678	1.006951	1.004476	82.27	83.30	83.85	84.47
0.2996	1.014208	1.011192	1.008450	1.005968	82.38	83.32	83.85	84.41
[tris-2-HEA][Ac]: 0.05 mol·kg^− 1^								
0.0000	1.002418	0.999698	0.997105	0.994773				
0.0488	1.004004	1.001238	0.998627	0.996271	82.40	83.48	83.98	84.61
0.0987	1.005608	1.002793	1.000179	0.997800	82.42	83.52	83.87	84.48
0.1497	1.007232	1.004390	1.001748	0.999350	82.45	83.41	83.87	84.44
0.1994	1.008821	1.005920	1.003267	1.000862	82.37	83.43	83.87	84.37
0.2472	1.010317	1.007399	1.004719	1.002301	82.41	83.36	83.85	84.33
0.3001	1.011983	1.009029	1.006307	1.003890	82.35	83.28	83.85	84.26
[tris-2-HEA][Ac]: 0.10 mol·kg^− 1^								
0.0000	1.005279	1.002501	0.999872	0.997523				
0.0500	1.006890	1.004071	1.001418	0.999047	82.54	83.49	84.11	84.68
0.1001	1.008489	1.005630	1.002962	1.000572	82.54	83.49	84.02	84.56
0.1499	1.010065	1.007163	1.004477	1.002072	82.53	83.50	84.02	84.53
0.1999	1.011611	1.008691	1.005988	1.003579	82.66	83.52	84.03	84.46
0.2491	1.013140	1.010201	1.007470	1.005042	82.66	83.45	84.00	84.45
0.2993	1.014686	1.011698	1.008948	1.006526	82.65	83.50	84.05	84.42
[tris-2-HEA][Ac]: 0.15 mol·kg^− 1^								
0.0000	1.008027	1.005184	1.002520	1.000136				
0.0498	1.009626	1.006736	1.004041	1.001624	82.46	83.54	84.30	85.09
0.0997	1.011234	1.008291	1.005560	1.003122	82.29	83.43	84.24	84.91
0.1494	1.012833	1.009836	1.007081	1.004605	82.16	83.33	84.08	84.83
0.1999	1.014453	1.011401	1.008619	1.006113	82.05	83.24	83.97	84.71
0.2500	1.016055	1.012955	1.010143	1.007607	81.96	83.14	83.87	84.61
0.3002	1.017687	1.014495	1.011654	1.009094	81.76	83.07	83.80	84.52

These data illustrate, that the density of the solutions increases with addition of the amino acid and PILs and decreases as temperature rising. apparent molar volumes of These solutions can be calculated by following Eq. 2 and their related value are given in Table 3:

1 $${V_\varphi }=\frac{M}{d} - \left[ {\frac{{\left( {d - {d_0}} \right)}}{{md{d_0}}}} \right]$$

**Fig. 2:**
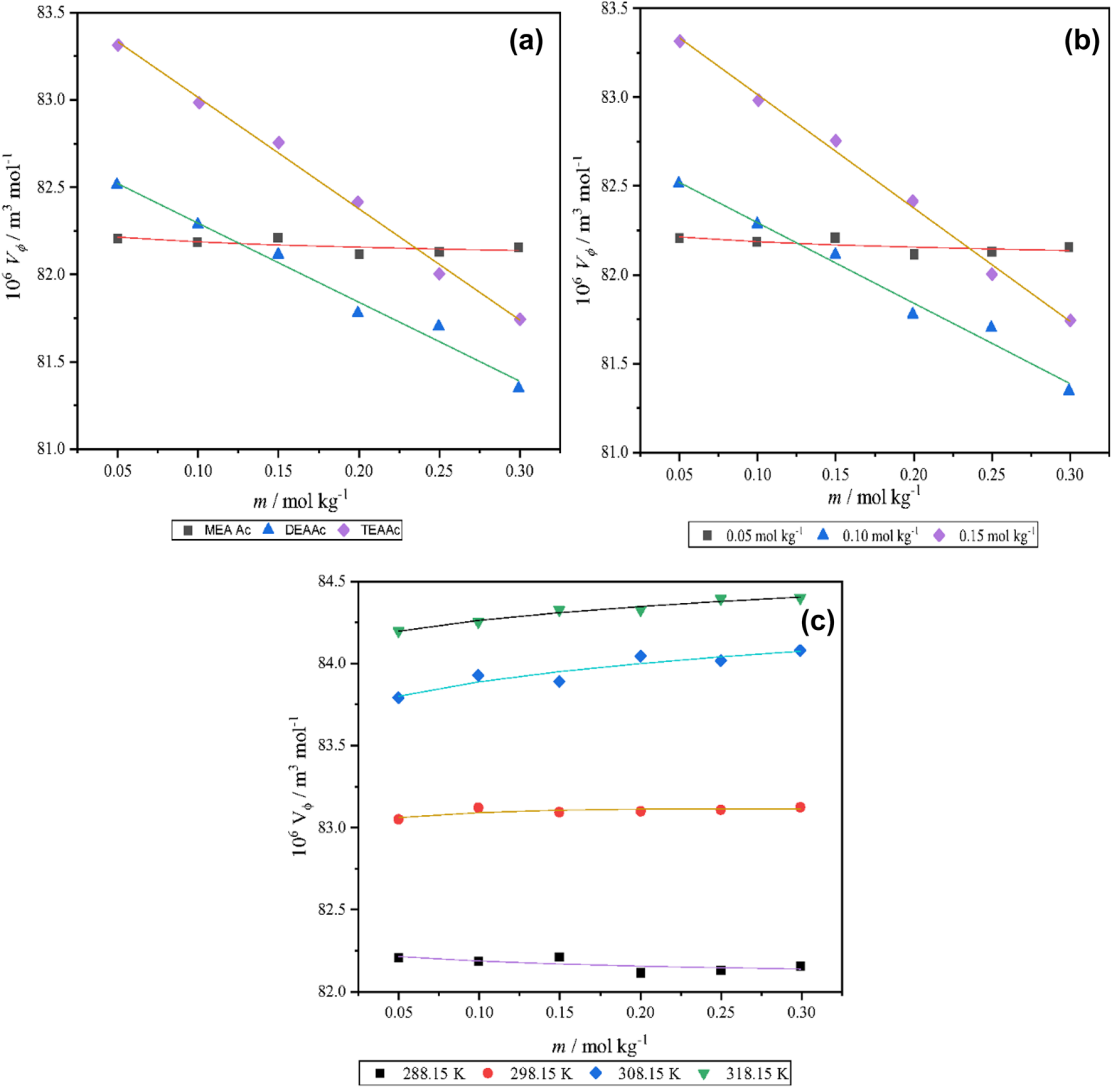
Variation of apparent molar volumes with (**a**) concentration of L-proline, (**b**) temperature, and (**c**) cation size of the ionic liquid.

Where, *M* represents the molar mass of amino acids (kg.mol^-1^), *d* and *d*_*0*_ are density of solution and pure solvent (kg.m^-3^), respectively and *m* is molality of experimental solution mol.kg^-1^. The thermodynamic properties of the studied solutions have been examined to achieve a good insight around them. An example of variation of the *V*_*φ*_ values by the molality of the L-proline in the presence of the studied PILs, their concentration and, the temperature has been illustrated in Fig. 2.

The calculated apparent molar properties have been correlated using Redlich-Mayer model as given by following Eq. (2):

2 $${V_\varphi }=V_{\varphi }^{0}+{S_v}m$$

where, $V_{\varphi }^{0}$, ${S_v}$ are given in Table 4, for the ternary aqueous solution of PILs. The $V_{\varphi }^{0}$ is the intercept of Eq. (2) which is called standard partial molar volume^22,23^.

**Table 4:** Parameters of the Redlich–Mayer equation and standard deviation of L-proline in aqueous PILs for the apparent molar volume at different temperatures.

System	*T*	$V_{\varphi }^{0}$	*S*_V_	*E*	$\alpha$	*Hc*	σ*V*_φ_
L-proline in aqueous solutions of [MEA][Ac] 0.05 mol·kg^− 1^	288.15	82.294 ± 0.029	−0.415 ± 0.015	0.072	8.769	−0.00095	0.029
	298.15	82.943 ± 0.021	0.682 ± 0.010	0.063	7.55		0.020
	308.15	83.559 ± 0.044	1.186 ± 0.023	0.053	6.352		0.043
	318.15	84.017 ± 0.020	0.873 ± 0.010	0.044	5.182		0.019
L-proline in aqueous solutions of [MEA][Ac] 0.1 mol·kg^− 1^	288.15	82.618 ± 0.057	0.713 ± 0.030	0.071	8.55	0.00093	0.056
	298.15	83.592 ± 0.036	0.849 ± 0.019	0.080	9.551		0.036
	308.15	84.104 ± 0.035	1.434 ± 0.018	0.089	10.587		0.035
	318.15	85.262 ± 0.033	1.007 ± 0.017	0.098	11.522		0.032
L-proline in aqueous solutions of [MEA][Ac] 0.15 mol·kg^− 1^	288.15	83.434 ± 0.043	1.2070 ± 0.022	0.166	19.914	−0.00631	0.042
	298.15	84.914 ± 0.038	−2.033 ± 0.019	0.103	12.136		0.037
	308.15	85.428 ± 0.037	−0.769 ± 0.019	0.040	4.676		0.037
	318.15	85.646 ± 0.019	−0.25 ± 0.097	−0.023	−2.703		0.018
L-proline in aqueous solutions of [DEA][Ac] 0.05 mol·kg^− 1^	288.15	82.092 ± 0.019	1.051 ± 0.009	0.142	17.282	−0.00291	0.018
	298.15	83.742 ± 0.022	−1.357 ± 0.011	0.113	13.466		0.021
	308.15	84.159 ± 0.031	1.91 ± 0.016	0.084	9.942		0.031
	318.15	85.227 ± 0.030	0.123 ± 0.015	0.055	6.402		0.030
L-proline in aqueous solutions of [DEA][Ac] 0.1 mol·kg^− 1^	288.15	82.626 ± 0.047	−1.42 ± 0.024	0.092	11.077	−0.00042	0.046
	298.15	83.788 ± 0.054	−3.579 ± 0.028	0.087	10.423		0.053
	308.15	84.239 ± 0.051	−1.282 ± 0.026	0.083	9.87		0.050
	318.15	85.317 ± 0.046	−2.687 ± 0.024	0.079	9.253		0.045
L-proline in aqueous solutions of [DEA][Ac] 0.15 mol·kg^− 1^	288.15	82.626 ± 0.047	−1.42 ± 0.025	0.090	10.851	−0.0002	0.047
	298.15	83.788 ± 0.043	−3.579 ± 0.022	0.088	10.472		0.042
	308.15	84.239 ± 0.025	−1.282 ± 0.013	0.086	10.165		0.025
	318.15	85.317 ± 0.034	−2.687 ± 0.018	0.084	9.807		0.034
L-proline in aqueous solutions of [TEA][Ac] 0.05 mol·kg^− 1^	288.15	82.202 ± 0.030	1.292 ± 0.015	0.129	15.692	−0.00266	0.022
	298.15	83.293 ± 0.037	1.478 ± 0.019	0.102	12.287		0.029
	308.15	84.281 ± 0.032	−1.808 ± 0.016	0.076	8.982		0.016
	318.15	84.84 ± 0.022	−1.134 ± 0.011	0.049	5.783		0.014
L-proline in aqueous solutions of [TEA][Ac] 0.1 mol·kg^− 1^	288.15	82.522 ± 0.035	−0.173 ± 0.018	0.110	13.307	−0.00165	0.035
	298.15	83.452 ± 0.024	0.255 ± 0.012	0.093	11.181		0.023
	308.15	84.43 ± 0.034	−1.975 ± 0.018	0.077	9.096		0.017
	318.15	85.031 ± 0.031	−1.931 ± 0.016	0.060	7.09		0.011
L-proline in aqueous solutions of [TEA][Ac] 0.15 mol·kg^− 1^	288.15	82.641 ± 0.030	−0.399 ± 0.015	0.103	12.448	−0.00077	0.029
	298.15	83.743 ± 0.012	−0.681 ± 0.006	0.095	11.361		0.418
	308.15	84.487 ± 0.024	−0.414 ± 0.012	0.087	10.345		0.479
	318.15	85.435 ± 0.026	−1.501 ± 0.013	0.080	9.325		0.500

The infinite dilution apparent molar expansibility $E_{\varphi }^{0}$ (cm^3^mol^− 1^K), isobaric thermal expansion coefficient (K^− 1^) and Helper constant values at different temperatures.

Possibly, amino-acids is surrounded by water molecules and the distance among them is relatively high, resulting in polar interactions with water molecules. The $V_{\varphi }^{0}$ value of the studied solutions are increased by raising of the concentration of PILs and the temperature, hence interactions are more powerful in the high temperatures and high concentration of PILs^24^. The ${S_v}$ value is the criteria of solute-solute interactions and the positive values of ${S_v}$ show that there is an interaction between L-proline molecules. The $V_{\varphi }^{0}$ values temperature dependency are fitted with a second-degree polynomial equation,^25^

3 $$V_{\varphi }^{0}=A+BT+C{T^2}$$

The empirical parameters of *A*, *B*, and *C* have been used to calculate the standard apparent molar expansibility at constant pressure $E_{\varphi }^{0}$ using following Eq. (2)^5^ .

4 $$E_{\varphi }^{0}={\left( {\frac{{\partial V_{\varphi }^{0}}}{{\partial T}}} \right)_p}=B+2CT$$

The values of $E_{\varphi }^{0}$ given in Table 4 shows that $E_{\varphi }^{0}$ is decreased by an increase in temperature. Based upon obtained result, it is assumed some of water molecules are being released from hydration layer when temperature increase. This increase in volume of L-proline is faster in the presence of [tris-2-HEA][Ac] rather than two other PILs^26^. For better understanding of this behavior the apparent isobaric thermal expansion was evaluated by the following equation^27^,

5 $$\alpha =\frac{{E_{\varphi }^{0}}}{{V_{\varphi }^{0}}}$$

The calculated values of *α* for amino acid are given in Table 4. This table indicates that the apparent isobaric thermal expansion values decrease by an increase in temperature due to the broken hydrogen bonds^28^. The structure making/breaking property of the solute (_L_-proline) in aqueous of (water + PILs) may be determined by helper s constant which is obtained by below Eq. (2)^9,29^

6 $${\left( {\frac{{\partial {C_P}}}{{\partial P}}} \right)_T}= - T{\left( {\frac{{{\partial ^2}V_{\varphi }^{0}}}{{\partial {T^2}}}} \right)_P}= - 2CT$$

where,$\:\:\left({\partial\:}^{2}{V}_{\phi\:}^{0}/{\partial\:T}^{2}\right)$ is the helper s constant for the L-proline in solutions (water + PIL) are given in Table 3. The positive or negative sign of this parameter determine the structure maker or structure breaker of solute in solution, respectively. Generally, positive values of this parameter mean the solute has structure maker behavior of solute (L-proline) in solution of (water + PILs). It could be deducted the H-bonding of the L-proline with water molecules would be affected by the presence of the studied PILs to rearrange the water molecules bind to L-proline and the PILs.

## Ultrasonic and compressibility properties of the bulk

The Newton-Laplace formula has been used (Eq. 7) to calculate the isentropic compressibility^30^. Furthermore, apparent molar isentropic compressibility of the amino acid in the PILs + water solutions were evaluated using the Eqs. (8), ^31^, also in this study isentropic compressibility and apparent molar isentropic compressibility related data are reported in Table 5.

7 $${\kappa _s}=\frac{1}{{d{u^2}}}$$

8 $${\kappa _\varphi }=\frac{{{\kappa _S}{d_0} - d{\kappa _{{S_0}}}}}{{md{d_0}}}+\frac{{{\kappa _S}M}}{d}$$

where, the symbols, *u*,* κ*_*s*_, *κ*_*s0*_ represents the speed of sound, isentropic compressibility of solution, and isentropic compressibility of solvent, respectively.

**Table 5:** Isentropic compressibility and apparent molar isentropic compressibility of ternary solutions in temperature range (298.15 to 318.15) K and concentration range (0.05 to 0.15) mol.kg^− 1^.

m	*u* / m s^− 1^				*κS* / Pa^-1^				*κφ* / m^3^ mol^-1^ Pa^-1^			
	288.15 K	298.15 K	308.15 K	318.15 K	288.15 K	298.15 K	308.15 K	318.15 K	288.15 K	298.15 K	308.15 K	318.15 K
[2−HEA][Ac]: 0.05 mol kg^− 1^												
0.0000	1471.72	1501.38	1523.80	1539.79	4.61	4.44	4.33	4.25	−3.38	−2.90	−2.33	−1.48
0.0497	1476.01	1505.36	1527.31	1543.00	4.58	4.41	4.30	4.22	−3.12	−2.49	−1.78	−1.36
0.0993	1480.21	1509.28	1530.65	1546.11	4.55	4.38	4.27	4.20	−3.06	−2.44	−1.67	−1.29
0.1496	1484.28	1512.79	1534.02	1549.21	4.51	4.36	4.25	4.18	−2.94	−2.24	−1.62	−1.25
0.2001	1488.49	1516.57	1537.50	1552.29	4.48	4.33	4.22	4.15	−2.93	−2.21	−1.62	−1.21
0.2499	1492.49	1520.12	1540.91	1555.26	4.45	4.30	4.20	4.13	−2.88	−2.15	−1.61	−1.17
0.2992	1496.49	1523.75	1544.67	1558.10	4.42	4.28	4.17	4.11	−2.84	−2.13	−1.67	−1.13
[2−HEA][Ac]: 0.10 mol kg^− 1^												
0.0000	1476.87	1505.98	1528.00	1543.66	4.58	4.41	4.30	4.22	−2.72	−2.31	−1.82	−1.28
0.0494	1480.92	1509.81	1531.38	1546.78	4.54	4.38	4.27	4.20	−2.76	−2.25	−1.58	−1.15
0.0997	1485.01	1513.65	1534.68	1549.83	4.51	4.35	4.25	4.17	−2.75	−2.23	−1.51	−1.09
0.1498	1489.31	1517.42	1538.09	1552.97	4.48	4.33	4.22	4.15	−2.84	−2.20	−1.53	−1.11
0.1993	1493.60	1521.34	1541.55	1555.89	4.44	4.30	4.20	4.13	−2.91	−2.26	−1.58	−1.08
0.2496	1497.59	1524.74	1544.82	1559.05	4.41	4.27	4.17	4.11	−2.86	−2.16	−1.55	−1.10
0.2994	1502.15	1528.72	1548.40	1561.91	4.38	4.24	4.15	4.09	−2.96	−2.22	−1.60	−1.07
[2−HEA][Ac]: 0.25 mol kg^− 1^												
0.0000	1481.89	1510.51	1532.12	1547.48	4.54	4.38	4.27	4.19	−2.65	−1.92	−1.54	−1.23
0.0501	1486.00	1514.20	1535.54	1550.64	4.51	4.35	4.24	4.17	−2.64	−1.91	−1.47	−1.12
0.1005	1490.03	1517.81	1538.85	1553.71	4.47	4.32	4.22	4.15	−2.60	−1.88	−1.41	−1.07
0.1501	1494.10	1521.52	1542.24	1556.71	4.44	4.30	4.19	4.13	−2.63	−1.93	−1.45	−1.06
0.1993	1498.11	1525.19	1545.51	1559.72	4.41	4.27	4.17	4.10	−2.65	−1.97	−1.46	−1.07
0.2500	1502.26	1528.99	1548.95	1562.65	4.38	4.24	4.14	4.08	−2.69	−2.00	−1.48	−1.05
0.3002	1506.04	1532.56	1552.14	1565.63	4.35	4.22	4.12	4.06	−2.64	−1.98	−1.46	−1.05
[bis−2−HEA][Ac]: 0.05 mol kg^− 1^												
0.0000	1472.55	1502.03	1524.29	1540.13	4.61	4.44	4.32	4.24	−3.48	−2.33	−1.98	−1.61
0.0500	1476.77	1505.85	1527.79	1543.41	4.57	4.41	4.30	4.22	−2.98	−2.22	−1.68	−1.32
0.1004	1480.95	1509.60	1531.15	1546.45	4.54	4.38	4.27	4.20	−2.92	−2.15	−1.58	−1.18
0.1494	1484.98	1513.28	1534.46	1549.57	4.51	4.35	4.24	4.17	−2.88	−2.14	−1.56	−1.20
0.1998	1489.16	1517.03	1537.89	1552.70	4.48	4.32	4.22	4.15	−2.87	−2.12	−1.56	−1.19
0.2489	1493.72	1520.32	1541.01	1555.33	4.44	4.30	4.20	4.13	−2.98	−2.02	−1.51	−1.09
0.3000	1498.21	1524.35	1544.58	1558.64	4.41	4.27	4.17	4.11	−3.00	−2.07	−1.53	−1.13
[bis−2−HEA][Ac]: 0.10 mol kg^− 1^												
0.0000	1478.65	1507.56	1529.31	1544.81	4.56	4.40	4.29	4.21	−3.26	−2.06	−2.20	−1.62
0.0498	1482.89	1511.33	1532.94	1548.04	4.53	4.37	4.26	4.19	−2.94	−2.15	−1.83	−1.28
0.1001	1486.99	1515.08	1536.52	1551.12	4.49	4.34	4.23	4.16	−2.83	−2.12	−1.78	−1.20
0.1496	1491.01	1518.94	1539.69	1554.18	4.46	4.31	4.21	4.14	−2.77	−2.18	−1.64	−1.18
0.1998	1495.06	1522.49	1542.92	1557.12	4.43	4.29	4.18	4.12	−2.73	−2.09	−1.57	−1.13
0.2494	1499.17	1526.17	1546.19	1560.20	4.40	4.26	4.16	4.10	−2.73	−2.08	−1.53	−1.14
0.2994	1503.06	1529.79	1549.54	1563.15	4.37	4.23	4.14	4.08	−2.67	−2.05	−1.53	−1.12
[bis−2−HEA][Ac]: 0.15 mol kg^− 1^												
0.0000	1484.42	1512.75	1534.07	1549.11	4.52	4.36	4.25	4.18	−2.30	−1.69	−1.13	−0.97
0.0498	1488.34	1516.32	1537.26	1552.11	4.49	4.33	4.23	4.16	−2.49	−1.85	−1.29	−0.98
0.1004	1492.42	1520.01	1540.66	1555.26	4.45	4.31	4.20	4.13	−2.55	−1.90	−1.39	−1.05
0.1501	1496.48	1523.73	1543.95	1558.15	4.42	4.28	4.18	4.11	−2.58	−1.94	−1.40	−1.00
0.1997	1500.53	1527.32	1547.26	1561.19	4.39	4.25	4.16	4.09	−2.59	−1.92	−1.41	−1.02
0.2495	1504.52	1530.92	1550.67	1564.28	4.36	4.23	4.13	4.07	−2.58	−1.91	−1.44	−1.04
0.2996	1508.51	1534.58	1554.09	1567.23	4.33	4.20	4.11	4.05	−2.55	−1.90	−1.45	−1.03
[tris−2−HEA][Ac]: 0.05 mol kg^− 1^												
0.0000	1475.29	1504.46	1526.47	1542.14	4.58	4.42	4.30	4.23	−3.05	−1.72	−1.54	−1.20
0.0488	1479.39	1508.06	1529.81	1545.21	4.55	4.39	4.28	4.20	−2.91	−2.04	−1.60	−1.20
0.0987	1483.44	1511.77	1532.99	1548.26	4.52	4.36	4.25	4.18	−2.81	−2.05	−1.48	−1.16
0.1497	1487.77	1515.79	1536.79	1551.59	4.49	4.33	4.23	4.16	−2.84	−2.14	−1.63	−1.22
0.1994	1491.67	1519.33	1540.04	1554.42	4.46	4.31	4.20	4.14	−2.76	−2.07	−1.58	−1.14
0.2472	1495.55	1523.14	1543.19	1557.56	4.43	4.28	4.18	4.11	−2.74	−2.13	−1.55	−1.18
0.3001	1499.86	1526.69	1546.67	1560.69	4.39	4.25	4.15	4.09	−2.73	−2.04	−1.53	−1.16
[tris−2−HEA][Ac]: 0.10 mol kg^− 1^												
0.0000	1484.03	1512.32	1533.61	1548.69	4.52	4.36	4.25	4.18	−2.96	−2.63	−1.98	−1.64
0.0500	1488.20	1516.20	1537.13	1551.95	4.48	4.33	4.23	4.16	−2.75	−2.18	−1.64	−1.27
0.1001	1492.27	1519.89	1540.45	1555.06	4.45	4.31	4.20	4.13	−2.68	−2.06	−1.53	−1.19
0.1499	1496.22	1523.49	1543.74	1557.92	4.42	4.28	4.18	4.11	−2.61	−1.99	−1.49	−1.08
0.1999	1500.30	1527.01	1547.13	1561.18	4.39	4.25	4.15	4.09	−2.59	−1.93	−1.48	−1.13
0.2491	1503.68	1530.88	1550.54	1564.23	4.37	4.22	4.13	4.07	−2.44	−1.98	−1.50	−1.13
0.2993	1508.01	1534.28	1553.58	1567.01	4.33	4.20	4.11	4.05	−2.50	−1.91	−1.42	−1.06
[tris−2−HEA][Ac]: 0.15 mol kg^− 1^												
0.0000	1492.35	1519.86	1540.46	1554.98	4.45	4.31	4.20	4.14	−2.23	−2.02	−1.60	−1.24
0.0498	1496.36	1523.51	1543.93	1558.11	4.42	4.28	4.18	4.11	−2.50	−1.87	−1.53	−1.07
0.0997	1500.33	1527.02	1547.21	1561.00	4.39	4.25	4.15	4.09	−2.48	−1.79	−1.42	−0.95
0.1494	1504.47	1530.51	1550.44	1564.04	4.36	4.23	4.13	4.07	−2.54	−1.76	−1.38	−0.96
0.1999	1508.57	1534.38	1553.90	1567.07	4.33	4.20	4.11	4.05	−2.55	−1.84	−1.41	−0.96
0.2500	1512.68	1538.05	1557.08	1570.08	4.30	4.17	4.08	4.03	−2.55	−1.84	−1.38	−0.96
0.3002	1516.17	1541.26	1559.69	1572.60	4.28	4.15	4.06	4.01	−2.45	−1.76	−1.25	−0.88

**Fig. 3:**
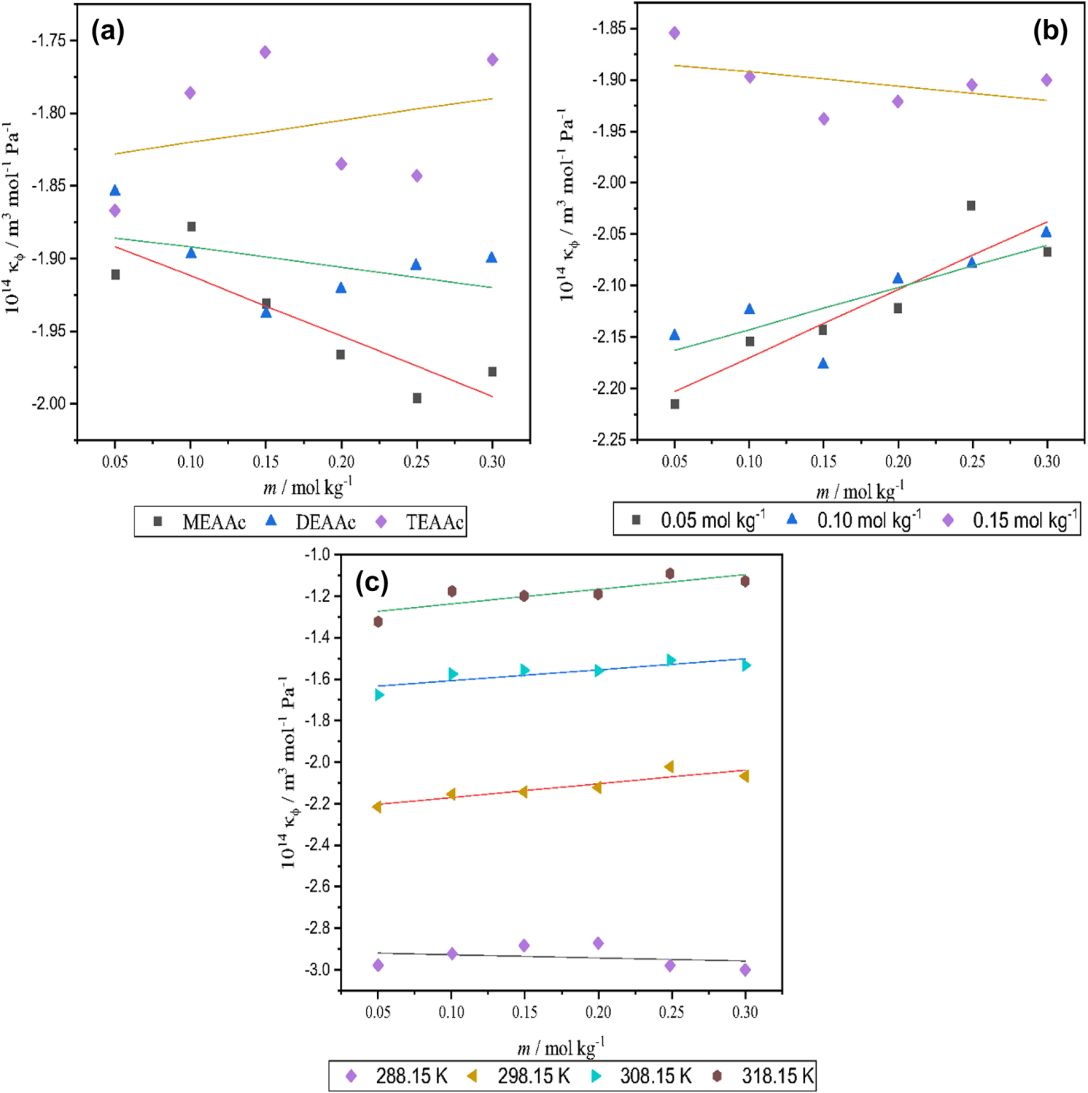
Variation of apparent molar isentropic compressibility *κ*_φ_with (**a**) concentration of L-proline, (**b**) temperature, and (**c**) cation size of the protic ionic liquid.

Analyzing of the *κ*_*s*_ shows an inverse relationship with amino acid concentration. The variation of the *κ*_*φ*_ values by the molality of the L-proline in the presence of the studied PILs, their concentration and, the temperature has been illustrated in Fig. 3.

This suggests that increasing amino acid concentration leads to a decrease in *κ*_*s*_ values. This phenomenon can be attributed to the disruption of the three-dimensional (3D) hydrogen bond network within water molecules. The breakdown of this network weakens the cohesive forces between water molecules, thereby reducing the overall resistance of the liquid to flow^32^. In order to calculate the standard partial molar isentropic compressibility of L-proline, Eq. (9) have been used:

9 $${\kappa _\varphi }=\kappa _{\varphi }^{0}+{S_k}m$$

where, $\kappa _{\varphi }^{0}$ is the partial molar isentropic compressibility and ${S_k}$ symbol is the empirical parameters of the Eq. (9). The obtained parameters for the investigated solutions are listed in Table 6 for the studied solutions.

**Table 6:** The values of *S*_*k*_ (m^3^.mol^− 3/2^.kg^1/2^.Pa^− 1^), $\kappa _{\varphi }^{0}$ (m^3^.mol^− 1^.Pa^− 1^) obtained for each mixture and standard deviation at the experimental temperatures from Eq. (7).

System	T	$\kappa _{\varphi }^{0}$	*S*_*k*_	σ$\kappa _{\varphi }^{{}}$
L−proline in aqueous solutions of [2-HEA][Ac] 0.05 mol·kg^− 1^	288.15	−3.376	1.229	0.016
	298.15	−2.899	1.955	0.037
	308.15	−2.333	3.345	0.01
	318.15	−1.479	0.512	0.004
L−proline in aqueous solutions of [2-HEA][Ac] 0.1 mol·kg^− 1^	288.15	−2.718	0.039	0.037
	298.15	−2.313	0.356	0.033
	308.15	−1.815	1.583	0.02
	318.15	−1.28	0.772	0.016
L−proline in aqueous solutions of [2-HEA][Ac] 0.15 mol·kg^− 1^	288.15	−2.653	0.213	0.024
	298.15	−1.92	0.274	0.022
	308.15	−1.536	0.537	0.02
	318.15	−1.226	0.647	0.009
L−proline in aqueous solutions of [bis-2-HEA][Ac] 0.05 mol·kg^− 1^	288.15	−3.483	3.157	0.053
	298.15	−2.331	0.525	0.029
	308.15	−1.98	1.776	0.031
	318.15	−1.614	1.695	0.044
L−proline in aqueous solutions of [bis-2-HEA][Ac] 0.1 mol·kg^− 1^	288.15	−3.256	1.736	0.03
	298.15	−2.006	−0.986	0.027
	308.15	−2.199	1.766	0.039
	318.15	−1.524	1.372	0.023
L−proline in aqueous solutions of [bis-2-HEA][Ac] 0.15 mol·kg^− 1^	288.15	−2.165	−1.953	0.029
	298.15	−1.591	−1.596	0.025
	308.15	−1.064	−1.316	0.023
	318.15	−0.883	−0.604	0.023
L−proline in aqueous solutions of [tris-2-HEA][Ac] 0.05 mol·kg^− 1^	288.15	−3.046	0.739	0.025
	298.15	−1.718	−1.904	0.035
	308.15	−1.542	−0.225	0.054
	318.15	−1.202	0.001	0.028
L−proline in aqueous solutions of [tris-2-HEA][Ac] 0.1 mol·kg^− 1^	288.15	−2.959	0.904	0.04
	298.15	−2.629	2.519	0.026
	308.15	−1.935	1.703	0.025
	318.15	−1.589	1.819	0.032
L−proline in aqueous solutions of [tris-2-HTEA][Ac] 0.15 mol·kg^− 1^	288.15	−2.228	−1.613	0.042
	298.15	−2.020	1.025	0.043
	308.15	−1.603	0.300	0.041
	318.15	−1.210	0.885	0.037

Negative value of this parameter means amino acid as solute are surrounded by PIL + water molecules as solvent so they are resisting against the compressibility more than the bulk. Also, the $\kappa _{\varphi }^{0}$ values decreased with rising temperature that related to the intrinsic expansion at higher temperature that pressure led to increase in volume at higher temperature.

## Kinematic and dynamic viscosity

In order to use protic ionic liquids in industry viscosity information is required. Viscosity is an important dynamic property of a fluid that affect other properties such as conductivity and resistance of flow. Accordingly, studying about this parameter is essential for fluid application in the industrial scale^33^. Viscosity gives us useful information about ionic hydration and ion-solvent interaction by the changing of temperature or concentration of aqueous solutions^34^. Dynamics viscosity and kinematic viscosity of (L-proline in (water + PIL) solution at (288.15 to 318.15) K is given in Table 7.

**Table 7:** Dynamic and kinetic viscosity of L-proline in PILs aqueous solutions at different temperatures.

*m*	*η*_*k*_				*η*_*D*_			
mol.kg^− 1^	mm^2^.S^− 1^				mPa.s			
	288.15 K	298.15 K	308.15 K	318.15 K	288.15 K	298.15 K	308.15 K	318.15 K
[2-HEA][Ac]: 0.05 mol·kg^− 1^								
0.0000	1.1532	0.9065	0.7393	0.6207	1.154	0.905	0.736	0.616
0.0497	1.1718	0.9190	0.7469	0.6262	1.175	0.919	0.745	0.623
0.0993	1.1885	0.9288	0.7579	0.6325	1.193	0.930	0.757	0.631
0.1496	1.2002	0.9411	0.7655	0.6392	1.207	0.944	0.766	0.641
0.2001	1.2148	0.9521	0.7734	0.6479	1.224	0.956	0.776	0.649
0.2499	1.2314	0.9639	0.7814	0.6553	1.242	0.970	0.784	0.657
0.2992	1.2455	0.9751	0.7899	0.6602	1.258	0.982	0.794	0.664
[2-HEA][Ac]: 0.10 mol·kg^− 1^								
0.0000	1.1726	0.9239	0.7544	0.6281	1.175	0.923	0.751	0.625
0.0494	1.1865	0.9306	0.7604	0.6332	1.191	0.932	0.759	0.630
0.0997	1.2040	0.944	0.7681	0.6408	1.211	0.946	0.768	0.639
0.1498	1.2187	0.9545	0.7788	0.6471	1.227	0.959	0.777	0.646
0.1993	1.2357	0.9676	0.7859	0.6547	1.246	0.973	0.786	0.655
0.2496	1.2561	0.9792	0.7972	0.6618	1.268	0.987	0.797	0.663
0.2994	1.2681	0.9929	0.8053	0.6702	1.283	1.002	0.808	0.671
[2-HEA][Ac]: 0.15 mol·kg^− 1^								
0.0000	1.1901	0.9337	0.7640	0.6371	1.194	0.934	0.763	0.638
0.0501	1.2092	0.9476	0.7739	0.6459	1.215	0.950	0.774	0.644
0.1005	1.2236	0.9594	0.7794	0.6521	1.232	0.963	0.782	0.652
0.1501	1.2408	0.9711	0.7880	0.6575	1.251	0.976	0.790	0.658
0.1993	1.2572	0.9836	0.7991	0.6645	1.270	0.990	0.801	0.665
0.2500	1.2725	0.9958	0.8058	0.671	1.287	1.004	0.810	0.673
0.3002	1.2899	1.0082	0.8154	0.6802	1.307	1.018	0.821	0.681
[bis-2-HEA][Ac]: 0.05 mol·kg^− 1^								
0.0000	1.1621	0.9126	0.7419	0.6216	1.164	0.911	0.739	0.619
0.0500	1.1792	0.9235	0.7505	0.6302	1.183	0.924	0.749	0.628
0.1004	1.1923	0.9353	0.7595	0.6358	1.198	0.937	0.759	0.636
0.1494	1.2125	0.9487	0.7705	0.6452	1.221	0.952	0.771	0.643
0.1998	1.2238	0.9587	0.7777	0.6508	1.233	0.963	0.779	0.649
0.2489	1.2376	0.9698	0.7874	0.6581	1.252	0.978	0.791	0.658
0.3000	1.2602	0.9879	0.8011	0.6678	1.275	0.995	0.804	0.667
[bis-2-HEA][Ac]: 0.10 mol·kg^− 1^								
0.0000	1.1896	0.9332	0.7589	0.6423	1.193	0.933	0.757	0.633
0.0498	1.2049	0.9446	0.7671	0.6478	1.211	0.946	0.766	0.643
0.1001	1.2217	0.9581	0.7780	0.6531	1.229	0.962	0.779	0.651
0.1496	1.2395	0.9720	0.7891	0.6587	1.249	0.977	0.789	0.658
0.1998	1.2549	0.9826	0.7964	0.6643	1.267	0.989	0.800	0.665
0.2494	1.2728	0.9956	0.8069	0.6719	1.287	1.004	0.811	0.674
0.2994	1.2875	1.0072	0.8149	0.6782	1.304	1.017	0.820	0.681
[bis-2-HEA][Ac]: 0.15 mol·kg^− 1^								
0.0000	1.2195	0.9531	0.7733	0.6475	1.225	0.953	0.771	0.643
0.0498	1.2313	0.9643	0.7824	0.6534	1.239	0.968	0.783	0.652
0.1004	1.2510	0.9793	0.7933	0.6611	1.261	0.984	0.795	0.661
0.1501	1.2657	0.9905	0.8020	0.6678	1.278	0.997	0.805	0.669
0.1997	1.2819	1.0005	0.8116	0.6771	1.295	1.009	0.816	0.678
0.2495	1.3025	1.0175	0.8225	0.6847	1.319	1.028	0.830	0.686
0.2996	1.3171	1.0283	0.8306	0.6909	1.336	1.040	0.838	0.694
[tris-2-HEA][Ac]: 0.05 mol·kg^− 1^								
0.0000	1.1834	0.9304	0.7585	0.6351	1.186	0.930	0.756	0.633
0.0488	1.1980	0.9393	0.7675	0.6430	1.203	0.940	0.767	0.641
0.0987	1.2122	0.9498	0.7724	0.6495	1.219	0.952	0.771	0.649
0.1497	1.2312	0.9633	0.7817	0.6537	1.240	0.968	0.783	0.652
0.1994	1.2449	0.9754	0.7909	0.6600	1.256	0.981	0.793	0.661
0.2472	1.2627	0.9882	0.8003	0.6667	1.276	0.995	0.804	0.668
0.3001	1.2746	0.9969	0.8071	0.6722	1.290	1.006	0.812	0.675
[tris-2-HEA][Ac]: 0.10 mol·kg^− 1^								
0.0000	1.2168	0.9621	0.7732	0.6502	1.223	0.956	0.773	0.649
0.0500	1.2348	0.9678	0.7849	0.6562	1.243	0.971	0.786	0.656
0.1001	1.2505	0.9791	0.7946	0.6687	1.261	0.984	0.796	0.669
0.1499	1.2693	0.9915	0.8031	0.6746	1.282	0.999	0.807	0.676
0.1999	1.2854	1.0045	0.8140	0.6826	1.300	1.013	0.819	0.685
0.2491	1.3020	1.0168	0.8278	0.6955	1.319	1.027	0.831	0.699
0.2993	1.3194	1.0297	0.8340	0.7028	1.339	1.042	0.841	0.707
[tris-2-HEA][Ac]: 0.15 mol·kg^− 1^								
0.0000	1.2568	0.9850	0.7989	0.6663	1.267	0.99	0.801	0.669
0.0498	1.2753	0.9971	0.8072	0.6739	1.288	1.004	0.810	0.675
0.0997	1.2925	1.0095	0.8171	0.6815	1.307	1.018	0.822	0.684
0.1494	1.3081	1.0228	0.8289	0.6929	1.325	1.033	0.835	0.696
0.1999	1.3335	1.0394	0.8408	0.7040	1.353	1.051	0.848	0.708
0.2500	1.3458	1.0504	0.8505	0.7102	1.367	1.064	0.859	0.716
0.3002	1.3587	1.0604	0.8560	0.7250	1.383	1.076	0.866	0.725

**Fig. 4:**
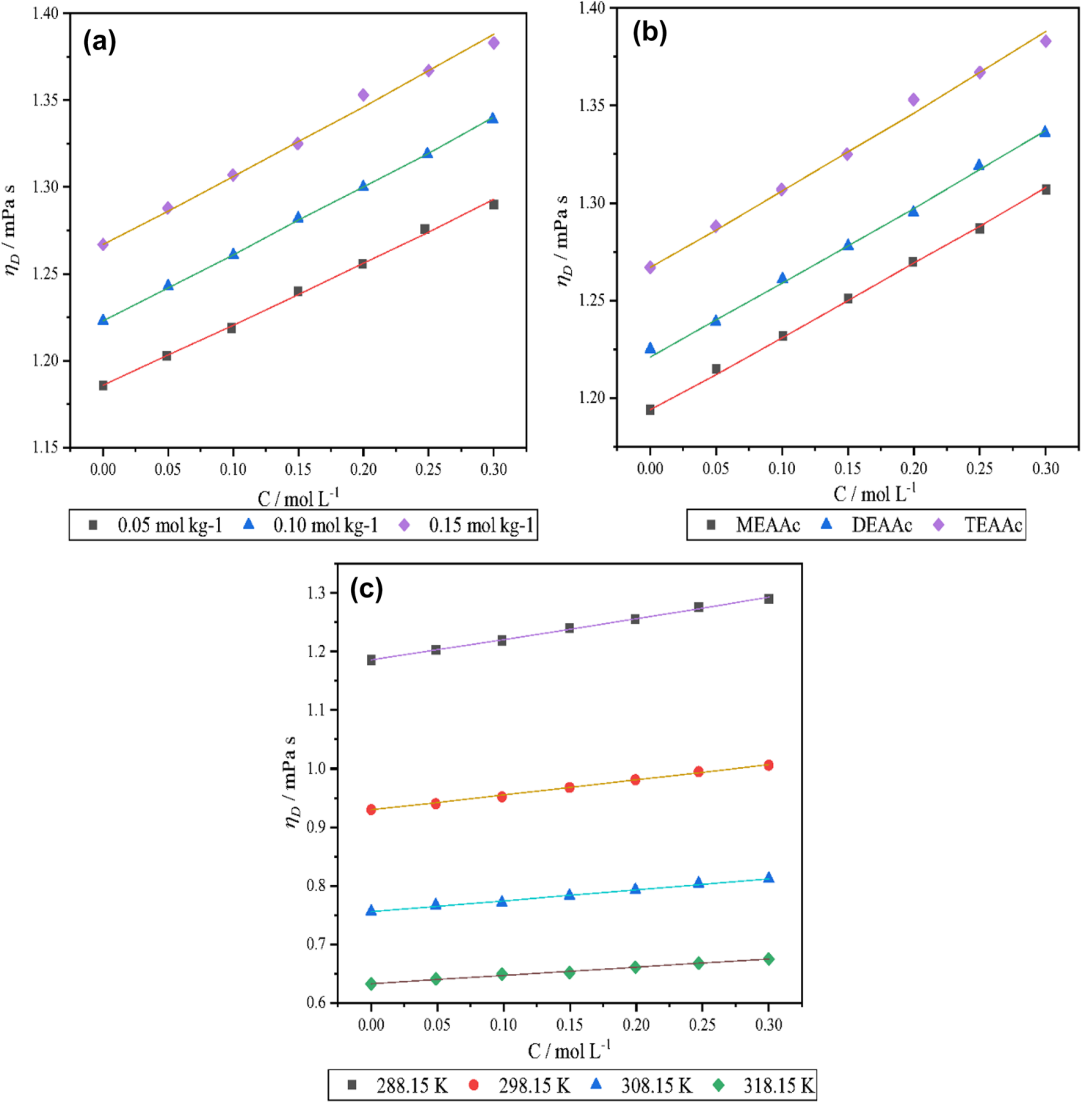
Variation of viscosity with (**a**) concentration of L-proline, (**b**) temperature, and (**c**) cation size of the ionic liquid.

Also, variation of *η* by the molality of the L-proline in the presence of the studied PILs, and, the temperature has been illustrated in Fig. 4.

As we can see in Table 7; Fig. 4 by increasing of concentration of L- proline and the PILs the amount of dynamic viscosity and kinetic viscosity of solutions are increased. Apparently, by the increase of molality of L-proline and the PILs, the number of collisions per volume are increased and the molecules lose their kinetic energy and they eager to get near each other, hence the solvent molecules cannot move rapidly because of strong interaction. Figuratively, the speed of liquids decreased and the viscosity is increased^35^. Viscosity of all of the systems under investigation exhibit a decreasing trend of variation with an increase in temperature. An increase in temperature may increase the kinetic energy of molecules, which in turn may decrease the hydrophilic- ionic and hydrophilic- hydrophobic interactions^36^. Furthermore, dynamic viscosity data has been fitted to the jones- dole equation:^37^

10 $$\:{\eta\:}_{r}=\frac{\eta\:}{{\eta\:}_{0}}=1+A\sqrt{m}+Bm$$

where, $\:\eta\:\:$is dynamic viscosity of solution and $\:{\eta\:}_{0\:}$is viscosity of solvent. The parameter *A* implies the long range columbic forces represents solute-solute interactions which is called is Falkenhagen coefficient The values of *A* coefficient usually is negligible for non-electrolyte solute^38^. Also, the values of *Sv* is another criterion for solute-solute interaction that show similar trend as *A* coefficient and was neglected. The viscosity *B*-coefficient is an empirical parameter that is a criterion of solute- solvent interaction. This parameter depends on the size and shape and structural effects induced by solute-solvent interaction^39^. The positive and large values of *B*-coefficient show a strong solute-solvent interaction which confirms the results of *Vφ*^0^ as another criterion of solute-solvent interaction. Also, based on Table 8.

**Table 8:** Viscosity *Ɓ*-coefficient values of ternary solutions obtained from Jones-Dole equation.

*T*	*B*
K	dm^3^·mol^− 1^
L-proline in aqueous solutions of [2-HEA][Ac] 0.05 mol·kg^− 1^	
288.15	0.297
298.15	0.277
308.15	0.258
318.15	0.256
L-proline in aqueous solutions of [2-HEA][Ac] 0.1 mol·kg^− 1^	
288.15	0.299
298.15	0.269
308.15	0.237
318.15	0.234
L-proline in aqueous solutions of [2-HEA][Ac] 0.15 mol·kg^− 1^	
288.15	0.307
298.15	0.292
308.15	0.242
318.15	0.213
L-proline in aqueous solutions of [bis-2-HEA][Ac] 0.05 mol·kg^− 1^	
288.15	0.301
298.15	0.29
308.15	0.277
318.15	0.248
L-proline in aqueous solutions of [bis-2-EA][Ac] 0.1 mol·kg^− 1^	
288.15	0.303
298.15	0.295
308.15	0.280
318.15	0.251
L-proline in aqueous solutions of [bis-2-HEA][Ac] 0.15 mol·kg^− 1^	
288.15	0.306
298.15	0.299
308.15	0.288
318.15	0.261
L-proline in aqueous solutions of [tris-2-HEA][Ac] 0.05 mol·kg^− 1^	
288.15	0.289
298.15	0.266
308.15	0.24
318.15	0.216
L-proline in aqueous solutions of [tris2-HEA][Ac] 0.1 mol·kg^− 1^	
288.15	0.309
298.15	0.292
308.15	0.290
318.15	0.288
L-proline in aqueous solutions of [tris-2-HEA][Ac] 0.15 mol·kg^− 1^	
288.15	0.306
298.15	0.287
308.15	0.272
318.15	0.270

*The viscosity B*-coefficients of all systems are decreased by increase of temperature. It refers to weakening of the solute-solvent interactions with the rising of temperature while the viscosity *B*-coefficient is bigger than *A* coefficient that implies the solute-solvent interactions are more powerful rather than solute-solute interaction.

### Refractive index and molar refraction

The refractive index of ternary solutions (L-proline + PILs + water) were measured in the temperature range of (288.15-318.15) K. all experimental values of refractive index of these solutions are given in Table 9. The tendency of the temperature dependences of refractive indexes is closely related to that of the density of these systems. All experimental density values of ternary solutions are given in Table 2. Table 9 represents the refractive index of the studied solution. based on this table, refractive index increased when the concentraion of amino acid get increased. however when the temperature is going up the refractive index get decreased and shows falling trend . The Lorentz–Lorenz was used to calculate the molar refraction:

11 $${R_M}=\frac{{n_{D}^{2} - 1}}{{n_{D}^{2}+2}} \cdot \frac{{{x_1} \cdot {M_1}+{x_2} \cdot {M_2}}}{d }$$

where, *x*_i_ and *M*_1_ and *M*_2_ are the mole fraction and molecular weight of each component of the mixture and d is the density of the solution. The values of ${R_M}$ and *n*_*D*_ are tabulated in Table 9.

**Table 9:** Refractive index and the molar refraction data of the aqueous solutions of L-proline in the presence of [2-HEA][Ac], [bis-2-HEA][Ac], and [tris-2-HEA][Ac] at different temperatures.

*m*	*n*_D_				*R*_M_			
mol kg^− 1^	288.15 K	298.15 K	308.15 K	318.15 K	288.15 K	298.15 K	308.15 K	318.15 K
[2-HEA][Ac]: 0.05 mol·kg^− 1^								
0.0000	1.3344	1.3332	1.3322	1.3311	3.7175	3.7153	3.7148	3.7132
0.0497	1.3355	1.3342	1.3333	1.3321	3.7404	3.7374	3.7380	3.7345
0.0993	1.3363	1.3352	1.3341	1.3328	3.7604	3.7596	3.7583	3.7538
0.1496	1.3372	1.3360	1.3351	1.3338	3.7816	3.7799	3.7808	3.7764
0.2001	1.3382	1.3371	1.3362	1.3348	3.8038	3.8034	3.8045	3.799
0.2499	1.3392	1.3379	1.337	1.3355	3.8259	3.8236	3.8249	3.8185
0.2992	1.3399	1.3387	1.3378	1.3363	3.8449	3.8438	3.8453	3.8388
[2-HEA][Ac]: 0.10 mol·kg^− 1^								
0.0000	1.3355	1.3340	1.3331	1.3320	3.7238	3.7194	3.7188	3.7167
0.0494	1.3363	1.3349	1.3339	1.3330	3.7437	3.7398	3.7395	3.7391
0.0997	1.3371	1.3359	1.3349	1.3338	3.7638	3.7621	3.7619	3.7598
0.1498	1.3379	1.3369	1.3359	1.3346	3.7839	3.7844	3.7843	3.7804
0.1993	1.3387	1.3379	1.3368	1.3353	3.8036	3.8064	3.8056	3.7997
0.2496	1.3396	1.3387	1.3376	1.3362	3.8247	3.8265	3.8259	3.8213
0.2994	1.3405	1.3394	1.3386	1.3370	3.8454	3.8453	3.8481	3.8417
[2-HEA][Ac]: 0.15 mol·kg^− 1^								
0.0000	1.3362	1.3346	1.3338	1.3327	3.7260	3.7201	3.7194	3.7191
0.0501	1.3372	1.3357	1.3346	1.3334	3.7483	3.7436	3.7422	3.7388
0.1005	1.3380	1.3366	1.3353	1.3342	3.7686	3.7650	3.7618	3.7594
0.1501	1.3389	1.3376	1.3363	1.3349	3.7896	3.7872	3.7842	3.7788
0.1993	1.3398	1.3385	1.3372	1.3358	3.8105	3.8083	3.8053	3.8000
0.2500	1.3404	1.3394	1.3382	1.3368	3.8284	3.8296	3.8278	3.8226
0.3002	1.3415	1.3406	1.3390	1.3375	3.8514	3.8540	3.8482	3.8420
[bis-2-HEA][Ac]: 0.05 mol·kg^− 1^								
0.0000	1.3348	1.3339	1.3327	1.3312	3.7210	3.7206	3.7183	3.7117
0.0500	1.3358	1.3346	1.3335	1.3319	3.7419	3.7401	3.7387	3.7312
0.1004	1.3364	1.3354	1.3344	1.3326	3.7601	3.7604	3.7603	3.7508
0.1494	1.3371	1.3364	1.3353	1.3334	3.7789	3.7825	3.7815	3.7710
0.1998	1.3381	1.3373	1.3363	1.3344	3.8011	3.8040	3.8041	3.7937
0.2489	1.3390	1.3382	1.3370	1.3351	3.8221	3.8252	3.8234	3.8129
0.3000	1.3398	1.3392	1.3377	1.3359	3.8424	3.8479	3.8431	3.8337
[bis-2-HEA][Ac]: 0.10 mol·kg^− 1^								
0.0000	1.3356	1.3347	1.3336	1.3324	3.7229	3.7226	3.7211	3.7196
0.0498	1.3366	1.3355	1.3346	1.3331	3.7435	3.7428	3.7434	3.7383
0.1001	1.3373	1.3365	1.3353	1.3339	3.7626	3.7651	3.7629	3.7588
0.1496	1.3381	1.3373	1.3361	1.3349	3.7827	3.7852	3.7831	3.7811
0.1998	1.3391	1.3379	1.3371	1.3357	3.8049	3.8034	3.8055	3.8016
0.2494	1.3401	1.3389	1.3380	1.3365	3.8270	3.8257	3.8270	3.8219
0.2994	1.3407	1.3397	1.3390	1.3374	3.8453	3.8460	3.8494	3.8433
[bis-2-HEA][Ac]: 0.15 mol·kg^− 1^								
0.0000	1.3368	1.3357	1.3343	1.3335	3.7274	3.7267	3.7239	3.7231
0.0498	1.3376	1.3365	1.3352	1.3344	3.7475	3.7470	3.7437	3.7446
0.1004	1.3384	1.3374	1.3359	1.3353	3.7677	3.7684	3.7632	3.7662
0.1501	1.3391	1.3383	1.3367	1.3361	3.7868	3.7896	3.7835	3.7867
0.1997	1.3401	1.3392	1.3377	1.3371	3.8089	3.8109	3.8059	3.8091
0.2495	1.3410	1.3402	1.3386	1.3380	3.8300	3.8333	3.8273	3.8305
0.2996	1.3420	1.3411	1.3393	1.3389	3.8524	3.8547	3.8467	3.8521
[tris-2-HEA][Ac]: 0.05 mol·kg^− 1^								
0.0000	1.3351	1.3340	1.3332	1.3323	3.7193	3.7182	3.7179	3.7175
0.0488	1.3360	1.3348	1.3339	1.333	3.7396	3.7378	3.7385	3.7381
0.0987	1.3366	1.3358	1.3347	1.3337	3.7577	3.7601	3.7587	3.7575
0.1497	1.3373	1.3367	1.3356	1.3346	3.7771	3.7816	3.7804	3.7792
0.1994	1.3380	1.3377	1.3365	1.3354	3.7961	3.8040	3.8017	3.7996
0.2472	1.3390	1.3386	1.3375	1.3364	3.8178	3.8248	3.8237	3.8216
0.3001	1.3401	1.3392	1.3385	1.3374	3.8417	3.8437	3.8469	3.8448
[tris-2-HEA][Ac]: 0.10 mol·kg^-1^								
0.0000	1.3372	1.3361	1.3349	1.3341	3.7400	3.7391	3.7378	3.7365
0.0500	1.3379	1.3370	1.3361	1.3351	3.7592	3.7605	3.7612	3.7600
0.1001	1.3386	1.3379	1.3369	1.3359	3.7784	3.7820	3.7817	3.7800
0.1499	1.3396	1.3388	1.3378	1.3368	3.8005	3.8031	3.8029	3.802
0.1999	1.3403	1.3397	1.3388	1.3375	3.8196	3.8245	3.8255	3.821
0.2491	1.3411	1.3406	1.3399	1.3384	3.8399	3.8459	3.8490	3.843
0.2993	1.3420	1.3415	1.3408	1.3394	3.8608	3.867	3.8703	3.865
[tris-2-HEA][Ac]: 0.15 mol·kg^-1^								
0.0000	1.3388	1.3379	1.3369	1.3358	3.7374	3.3767	3.7358	3.7344
0.0498	1.3398	1.3389	1.3377	1.3367	3.7572	3.7590	3.7570	3.7559
0.0997	1.3407	1.3398	1.3386	1.3377	3.7783	3.7803	3.7785	3.7785
0.1494	1.3413	1.3408	1.3395	1.3385	3.7964	3.8026	3.7998	3.7990
0.1999	1.3422	1.3417	1.3405	1.3392	3.8176	3.8241	3.8224	3.8187
0.2500	1.3432	1.3425	1.3414	1.3398	3.8398	3.8444	3.8439	3.8372
0.3002	1.3441	1.3334	1.3423	1.3407	3.8608	3.7637	3.8654	3.8589

Molar refraction is useful parameter related to solute–solvent interactions and molecular polarizability in the solution. Based on Table 9 it is clear that the ${R_M}$ values increase with increasing the amount of ionic liquid which indicating high polarizability in the solutions studied and strong interactions between PILs and L-proline.

### Hydration behavior interpretation

In early research stages, it is mentioned that L-proline has the smallest cavity volume and surface area compared to the other molecules. This suggests that L-proline is a more compact solute, implying that its hydration behavior may be different from the other molecules. The smaller cavity volume and surface area indicate that L-proline may have fewer available sites for H-bonding with solvent molecules. Additionally, the dielectric (hydration) energy of L-proline is mentioned to be the least negative among the studied molecules. This indicates a weaker interaction between L-proline and the solvent compared to the other molecules. In the context of H-bonding, a less negative dielectric energy suggests that the H-bonding interactions between L-proline and solvent molecules may be weaker or less favorable compared to the other molecules.

On the other hand, [2-HEA][Ac], [bis-2-HEA][Ac], and [tris-2-HEA][Ac] are described as having progressively larger cavity volumes and surface areas. This implies that these molecules occupy more space and have more intricate interactions with the solvent compared to L-proline. Furthermore, these molecules exhibit more negative dielectric (hydration) energies, indicating stronger favorable interactions with the solvent. In terms of H-bonding, the larger cavity volumes and surface areas of these molecules provide more opportunities for H-bond formation with solvent molecules, leading to stronger and more favorable H-bonding interactions.

The temperature dependency of the hydration behavior is also mentioned. The apparent molar expansibility and apparent isobaric thermal expansion values decrease with increasing temperature. This suggests that as the temperature increases, the H-bonding interactions between L-proline and water molecules weaken, leading to the release of some water molecules from the hydration layer. This behavior may be attributed to the breaking of hydrogen bonds between L-proline and water molecules with increasing temperature.

Furthermore, the helper’s constant is discussed as a parameter that determines the structure-making or structure-breaking behavior of L-proline in the presence of the studied PILs. The positive or negative sign of the helper’s constant indicates whether L-proline acts as a structure maker or structure breaker in the solution. Positive values suggest that L-proline has a structure-making behavior, implying that it can rearrange the H-bonding network between water molecules and the PILs. This suggests that the presence of the studied PILs can influence the H-bonding interactions between L-proline and water molecules, potentially altering the hydration behavior.

In summary, the provided results suggest that L-proline exhibits a different hydration behavior compared to the studied molecules ([2-HEA][Ac], [bis-2-HEA][Ac], and [tris-2-HEA][Ac]). L-proline’s smaller cavity volume and surface area, along with its less negative dielectric energy, indicate a more compact solute with weaker H-bonding interactions with the solvent. On the other hand, the studied molecules have larger cavity volumes and surface areas, stronger dielectric energies, and can form more intricate H-bonding interactions with the solvent. The temperature dependency and the influence of the studied PILs further highlight the role of H-bonding in the hydration behavior of L-proline.

## Conclusion

The study suggests that L-proline has a weaker interaction with water molecules compared to the surrounding PILs ([2-HEA][Ac], [bis-2-HEA][Ac], and [tris-2-HEA][Ac]) due to its more compacted structure and less negative dielectric energy. These PILs, with their larger size and more intricate structures, interact more significantly strong with water molecules likely through hydrogen bonding. As temperature increases, the hydration layer around L-proline appears to be affected, with more water molecules released compared to the PIL solutions. This effect is more pronounced with [tris-2-HEA][Ac], possibly due to its larger size and more complex structure allowing it to compete more effectively with L-proline for hydrogen bonding with water molecules. The positive helper’s constant indicates that L-proline influences the surrounding water molecules to form a more ordered structure. However, the PILs might also be disrupting the H-bonding between L-proline and water by rearranging the water molecules around L-proline and forming their own hydrogen bonds with the water.

## Electronic supplementary material

Below is the link to the electronic supplementary material.

Supplementary Material 1

## Data Availability

All data generated or analyzed during this study are included in this published article [and its supplementary information files].

## Abbreviations

PIL: Protic ionic liquid
[2-HEA][Ac]: 2-hydroxyethylammonium acetate
[bis-2-HEA][Ac]: bis-(2-hydroxyethylammonium) acetate
[tris-2-HEA][Ac]: tris-(2-hydroxyethylammonium) acetate
H-bonding: Hydrogen bonding
IL: Ionic liquid
