# Supplementary material for: Hydration behavior of L-proline in the presence of mono, bis, tris-(2-hydroxyethyl) ammonium acetate protic ionic liquids: Thermophysical properties
Source: Sci Rep. 2024 Nov 8;14:27229. doi: 10.1038/s41598-024-77341-6 (PMC11549441; doi:10.1038/s41598-024-77341-6)
Supplement: Supplementary file 1 — Supplementary Material 1 [file 41598_2024_77341_MOESM1_ESM.docx]

**“Supporting Information”**

**Hydration behavior of L-proline in the presence of mono, bis, tris (2-hydroxyethyl) ammonium acetate with thermophysical properties**

Mohammad Amin Morsali, Hemayat Shekaari*, Behrang Golmohammadi

*Department of Physical Chemistry, Faculty of Chemistry, University of Tabriz, Tabriz, Iran*

*Corresponding author Tel.: +98-41-33393139. Fax: +98-41-33340191.

Email: [hemayatt@yahoo.com](mailto:hemayatt@yahoo.com)

**
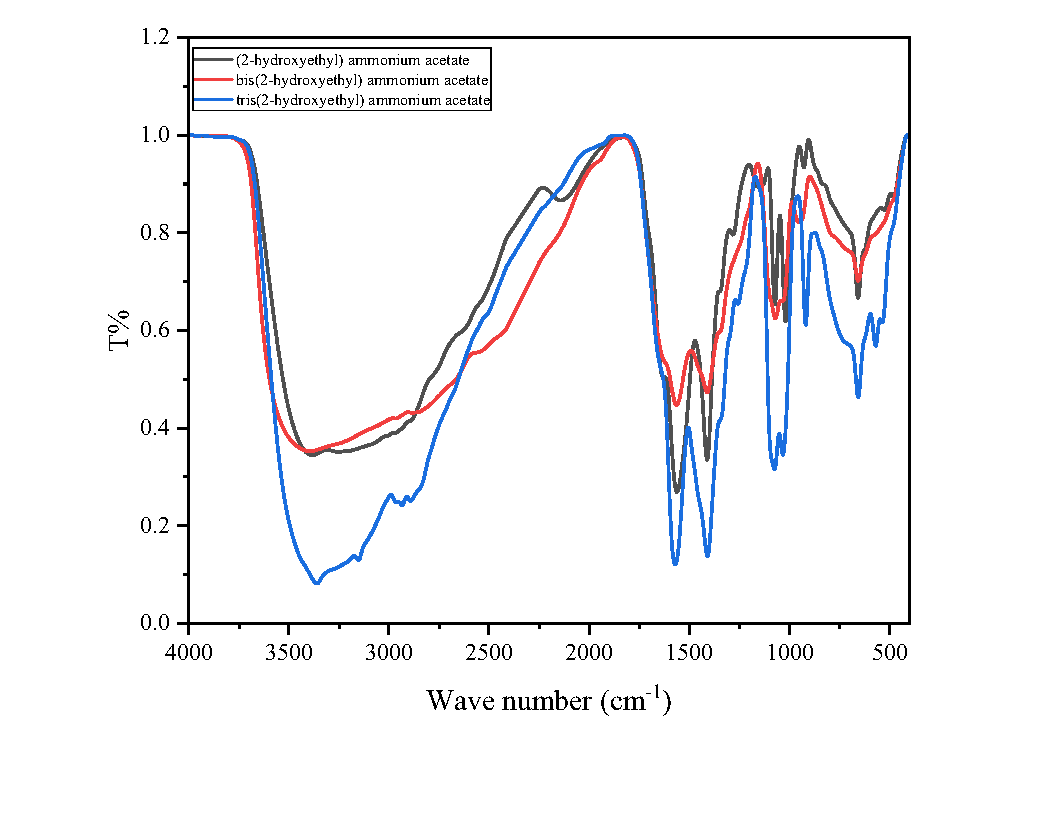
**

**Figure S1.** The FT-IR spectra of the PILs (2-hydroxyethyl) ammonium acetate ([2-HEA][Ac]), bis (2-hydroxyethyl) ammonium acetate [bis-2-HEA][Ac], tris (2-hydroxyethyl) ammonium acetate [tris-2-HEA][Ac].


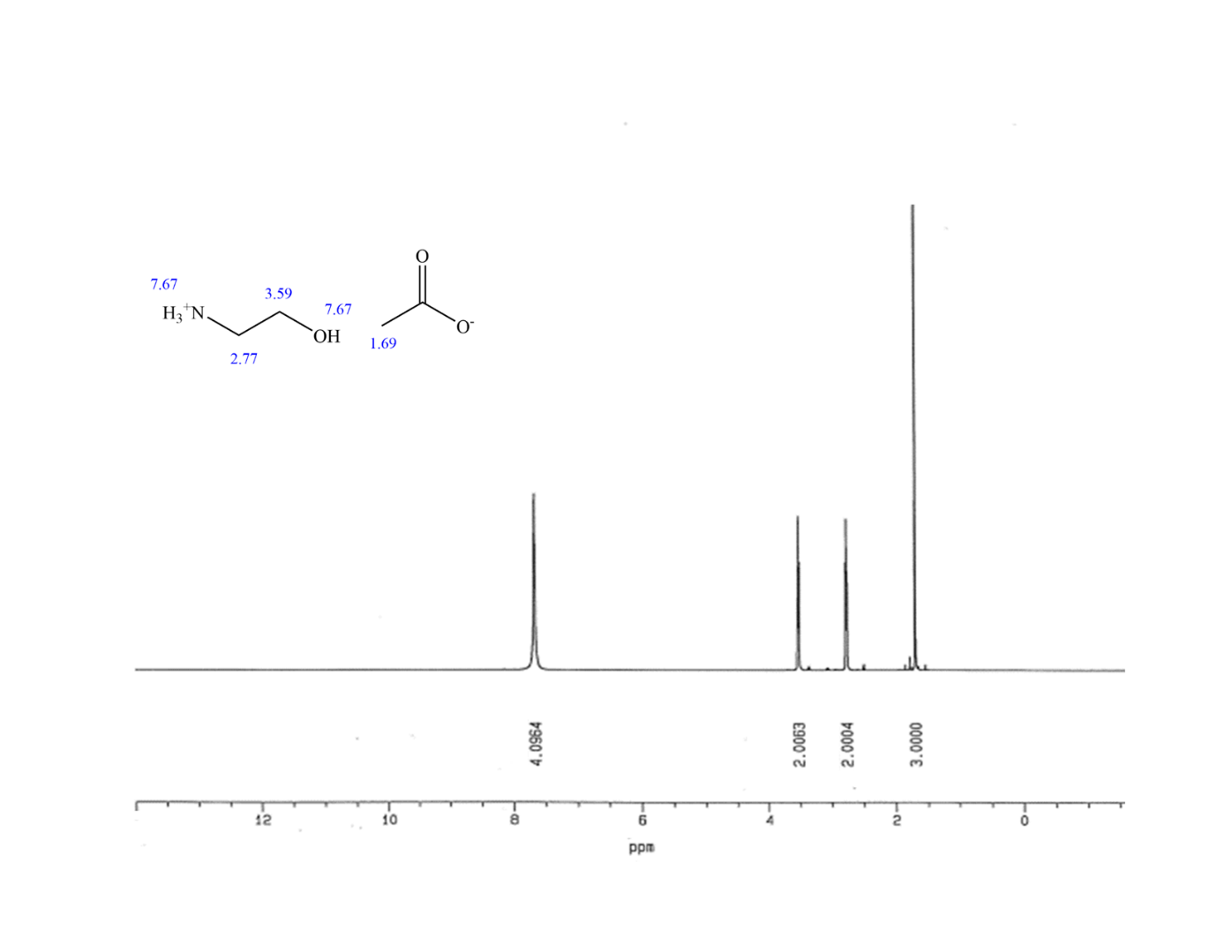


**Figure. S2.** ^1^HNMR spectrum of the synthesized PIL [2-HEA][Ac].


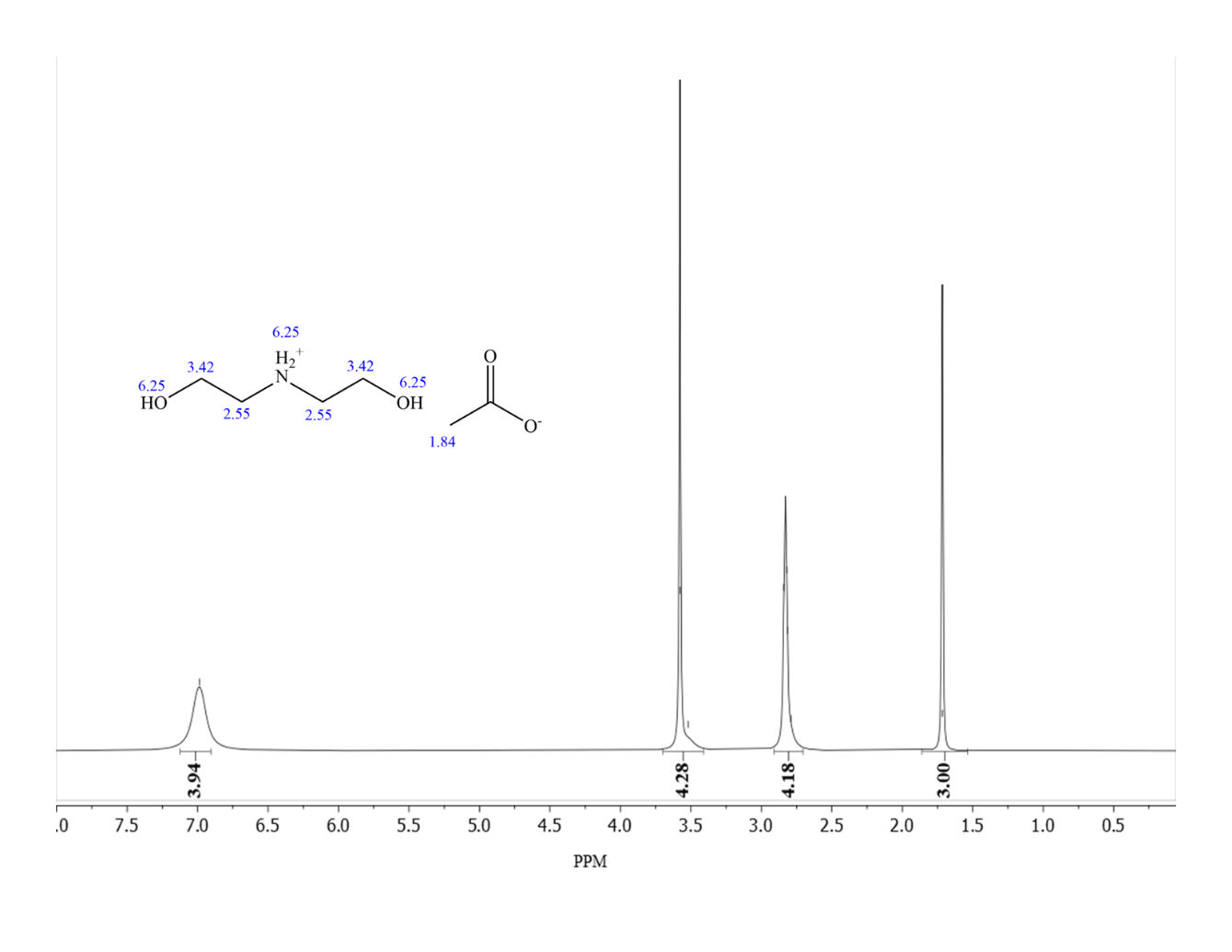


**Figure. S3.** ^1^HNMR spectrum of the synthesized PIL [bis-2-HEA][Ac].


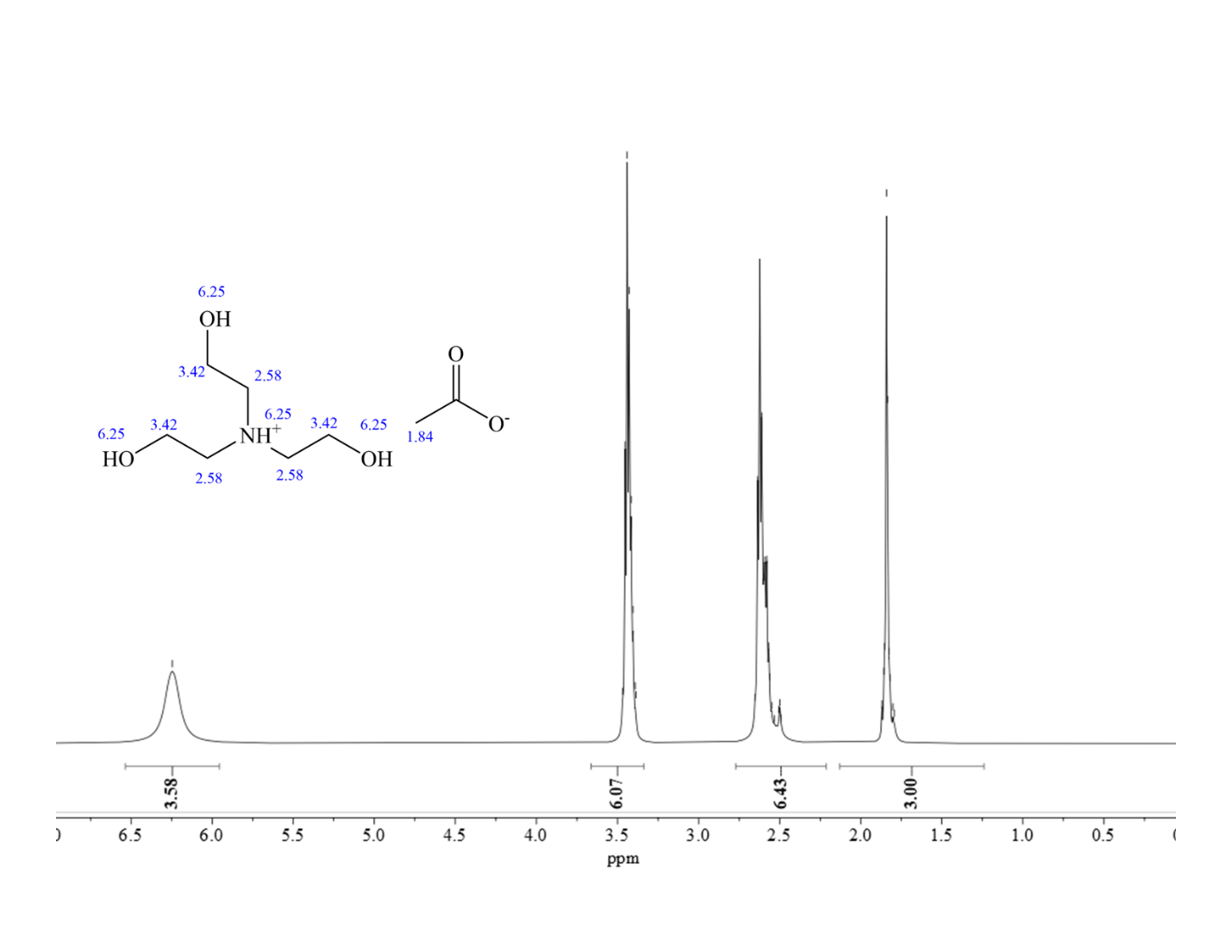


**Figure. S4.** ^1^HNMR spectrum of the synthesized PIL [tris-2-HEA][Ac].
